# Imaging in Hemophagocytic Lymphohistiocytosis: A Comprehensive Pictorial Essay

**DOI:** 10.7759/cureus.89203

**Published:** 2025-08-01

**Authors:** Caleb Bhatnagar, Emad Allam, Mathew Illimoottil, Sarah Illimoottil, Eric Errampalli, Sriya Kosaraju

**Affiliations:** 1 Radiology, Loyola University Medical Center, Maywood, USA; 2 Radiology, University of Missouri Kansas City School of Medicine, Kansas City, USA; 3 Internal Medicine, University of Missouri Kansas City School of Medicine, Kansas City, USA

**Keywords:** epstein barr virus (ebv), familial hlh (fhl), hemophagocytic lymphohistiocytosis (hlh), secondary cns, secondary hemophagocytic lymphohistiocytosis (hlh)

## Abstract

Hemophagocytic lymphohistiocytosis (HLH) is a rare but life-threatening condition characterized by excessive immune activation, resulting in severe systemic inflammation and multiorgan failure. This pictorial essay presents an overview of HLH imaging findings, accompanied by a discussion of pathophysiology, diagnosis, and treatment approaches. Imaging is crucial in diagnosing and monitoring HLH by identifying key features, such as splenomegaly, lymphadenopathy, and central nervous system (CNS) involvement. While the imaging findings associated with HLH are nonspecific and do not constitute part of the diagnostic criteria for HLH, they are helpful in the diagnostic process and can offer valuable insights into the extent of organ involvement and disease progression, guiding timely interventions. This article emphasizes the importance of recognizing imaging patterns associated with HLH for early diagnosis and effective management.

## Introduction and background

Hemophagocytic lymphohistiocytosis (HLH) is a rare but potentially fatal condition caused by excessive immune activation [1]. It stems from the defective function of natural killer (NK) cells and cytotoxic T lymphocytes and leads to uncontrolled proliferation of activated lymphocytes and macrophages. This immune response aggressively attacks red blood cells and leads to hallmark symptoms of fever, hepatosplenomegaly, and cytopenias [2]. HLH can be primary, due to genetic mutations and inborn errors of immunity, or secondary to infections, malignancies, or autoimmune disorders [3]. The true epidemiology of HLH, while difficult to assess, has been estimated to be one in 100,000 in children younger than 18 years of age for all ethnicities and races [4,5]. In Sweden, where the disease has been studied more extensively, the incidence of familial HLH (FHL) is estimated to be one in 50,000 births [6,7]. Understanding HLH's pathophysiology is important for recognizing its imaging manifestations. Accurate identification and monitoring through imaging are essential for timely intervention and management, which is critical given the rapidly progressive nature of HLH. In this article, we will discuss the etiology, pathophysiology, and treatment of HLH and explore the role of imaging in its diagnosis and presentation. 

Primary HLH, also known as FHL, is inherited in an autosomal recessive manner [8]. Caused by genetic mutations in the immune system, there is uncontrolled stimulation and impaired down-regulation of histiocytes, cytotoxic T cells, and NK cells, resulting in hypercytokinemia and systemic inflammatory response syndrome (SIRS) [8]. Some of the identified genes involved are the PRF1, UNC13D, and STXBP2 genes [9]. Primary HLH presents early in life, typically with symptoms starting in the first months to years of life [10]. 

Other related disorders caused by different mutations may present with HLH-like symptoms [11]. Flow cytometric testing can screen for protein deficiencies like perforin, XIAP, and SAP [12]. A CD107a assay can detect patients with defects in genes involved in degranulation, indicating specific genetic forms of HLH [13]. 

Secondary HLH does not have predisposing genetic factors and occurs due to external factors that trigger the body’s immune system. Commonly known triggers are infection (particularly Epstein-Barr virus (EBV) and other viruses), malignancy, individuals with autoimmune conditions, and pre-existing inflammatory conditions. This form of HLH tends to affect older patients and typically presents as an acute onset of symptoms after an external trigger [11]. The range in etiology is reflected in the way clinical symptoms present, making the diagnosis of secondary HLH challenging [14]. 

## Review

Pathophysiology 

HLH is a severe immune dysregulation that results in the uncontrolled activation and proliferation of immune cells, particularly macrophages and cytotoxic T cells. This pathological process triggers an excessive inflammatory response known as a cytokine storm, which is critical to the pathogenesis and clinical manifestations of the disease [15]. The normal regulatory mechanisms that control the activation and proliferation of immune cells are altered, with cytotoxic T cells and NK cells failing to function properly [16]. This defect is often due to mutations in genes responsible for the degranulation process and cytolytic function of these cells, such as PRF1, UNC13D, and STXBP2 in primary HLH [12,17]. As a result, these cells are unable to effectively kill target cells, leading to persistent activation [18].

Uncontrolled activation of cytotoxic T cells and macrophages results in excessive release of inflammatory cytokines, including interferon gamma (IFN-γ), tumor necrosis factor alpha (TNF-α), interleukin-6 (IL-6), and interleukin-1 beta (IL-1β) [19]. This cytokine storm perpetuates the cycle of inflammation and immune cell activation. Elevated cytokine levels lead to systemic inflammation, resulting in tissue damage and multi-organ dysfunction [20].

Hemophagocytosis, the process by which activated macrophages engulf and destroy blood cells, including red blood cells, white blood cells, and platelets, is the hallmark pathological finding of HLH but is not always detectable [1,21]. Its rate of detection in the bone marrow of HLH patients ranges from 25% to 100% [22]. This is because hemophagocytosis is neither sensitive nor specific [22]. Hemophagocytosis associated with HLH results in clinical symptoms of cytopenia, manifested by anemia, leukopenia, and thrombocytopenia [22]. The cascade of immune activation and cytokine release leads to widespread tissue and organ damage [23]. The main organs affected are the liver and spleen, where hepatosplenomegaly is common due to increased infiltration and phagocytic activity of activated immune cells [24]. The bone marrow is infiltrated by activated macrophages, which impair hematopoiesis (blood cell formation), resulting in cytopenias [25]. Central nervous system (CNS) involvement can cause neurologic symptoms such as seizures, ataxia, and altered mental status [26]. Lung involvement can result in interstitial pneumonitis, acute respiratory distress syndrome (ARDS), and pleural effusions. In severe cases, renal and cardiac function may be compromised by systemic inflammatory responses and direct organ infiltration of immune cells.

Diagnosis

Early recognition and diagnosis of HLH are paramount due to its potentially life-threatening damage to tissue and consequential multiorgan failure. Although rare, patients who present with the hallmark symptoms should be suspected of having the condition and thus should undergo diagnostic testing to initiate prompt treatment if needed. Diagnostic criteria consist of a genetic defect consistent with primary HLH or five of eight of the following symptoms and lab values: fever, splenomegaly, cytopenia in ≥ 2 cell lineages, hypertriglyceridemia and/or hypofibrinogenemia, hyperferritinemia, elevated soluble CD25, hemophagocytosis in bone marrow, spleen, lymph nodes, or liver, and low or absent NK-cell cytotoxicity [22].

Role of imaging in hemophagocytic lymphohistiocytosis

Imaging is not a specific part of the diagnostic criteria for HLH, although imaging can reveal or confirm splenomegaly, one of the diagnostic criteria [22,27]. However, it can be useful in the identification of potential underlying triggers or for understanding the scope of organ involvement [27]. Neuroimaging is important, as CNS involvement may necessitate intrathecal therapy [28].

Imaging findings in HLH are nonspecific in isolation and may mimic other conditions, such as ARDS in the chest or infectious, inflammatory, and neoplastic disorders in other areas [29]. Rapid progression and regression of findings post-therapy are typical [30]. 

Ethics statement regarding imaging

Informed consent was obtained from all patients to share the images included in this review. 

Chest imaging

**Figure 1:**
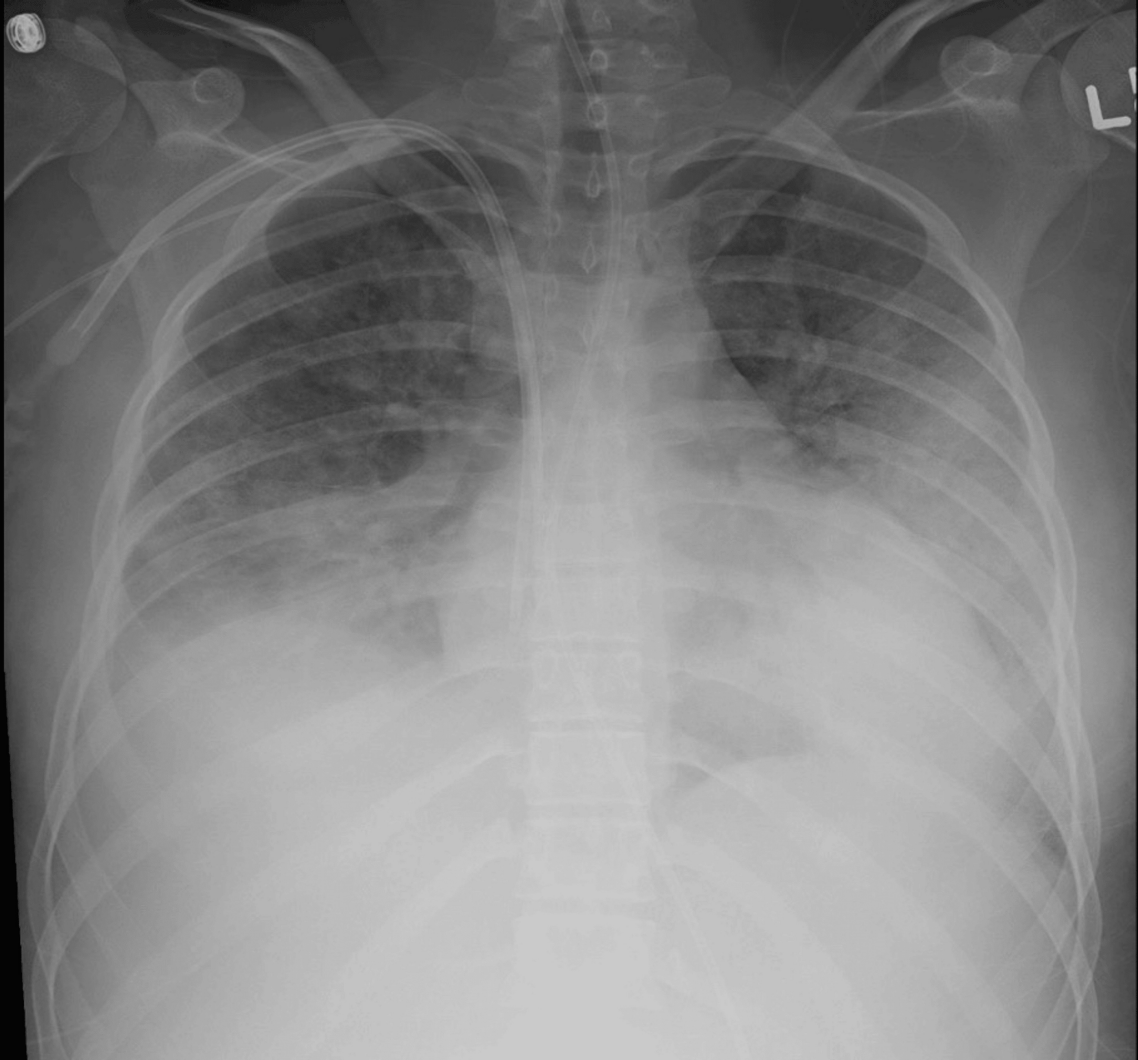
A 16-year-old child with lymphoma and secondary HLH Chest radiograph demonstrates bilateral alveolar and interstitial opacities and right pleural effusion. Right-sided venous access catheters have been placed for treatment. HLH: hemophagocytic lymphohistiocytosis

Chest radiography and CT are useful for assessment of pulmonary involvement in HLH [29]. As seen in Figure 1, patients may present with pulmonary alveolar/interstitial opacities with pleural effusions [29]. This corresponds to inflammation or infection within the lung parenchyma [29]. Lymphadenopathy also typically occurs due to uncontrolled immune activation with HLH [31]. 

Abdominal imaging 

**Figure 2:**
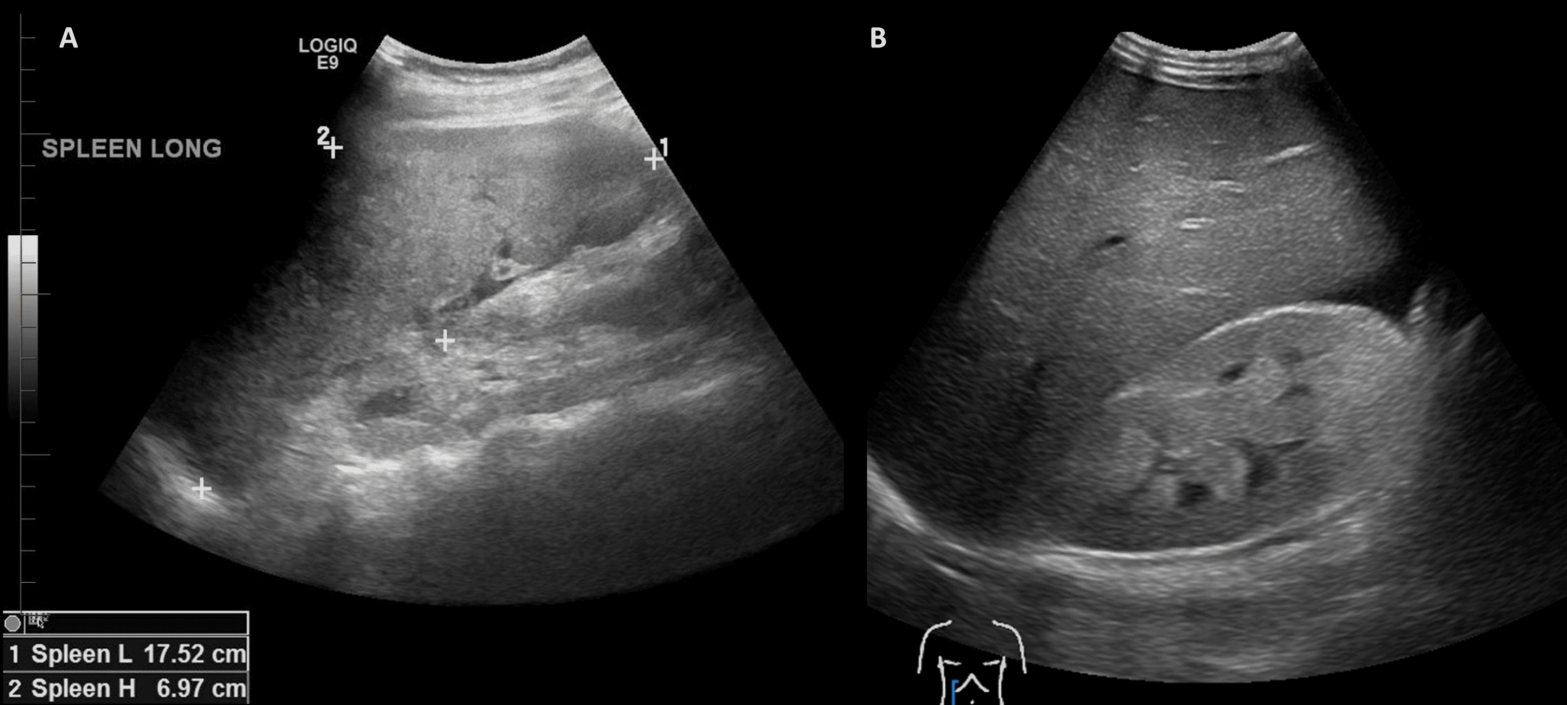
Abdominal ultrasound Abdominal ultrasound with corresponding symptoms seen in HLH. Images show splenomegaly (A) and hyperechoic kidneys (B). HLH: hemophagocytic lymphohistiocytosis

Ultrasound, CT, and MRI can be employed for the diagnosis of HLH [29]. Figure 2 below shows an abdominal ultrasound with relevant findings in HLH.

Given its wide availability and real-time results, ultrasound is typically used first for the evaluation of abdominal symptoms in HLH patients [32-34]. It can quickly detect enlargement of the liver or spleen, indicative of immune cell infiltration and increased phagocytic activity [35].

Hepatosplenomegaly is one of the hallmark symptoms of HLH and can be identified as uniformly enlarged, hypoechoic organs on US [36]. Severe systemic inflammation can cause ascites, which can be visualized on abdominal US as free fluid [37]. Lymphadenopathy can also be identified on US, typically appearing as enlarged hypoechoic nodes in the para-aortic, iliac, and mesenteric regions [38]. 

**Figure 3:**
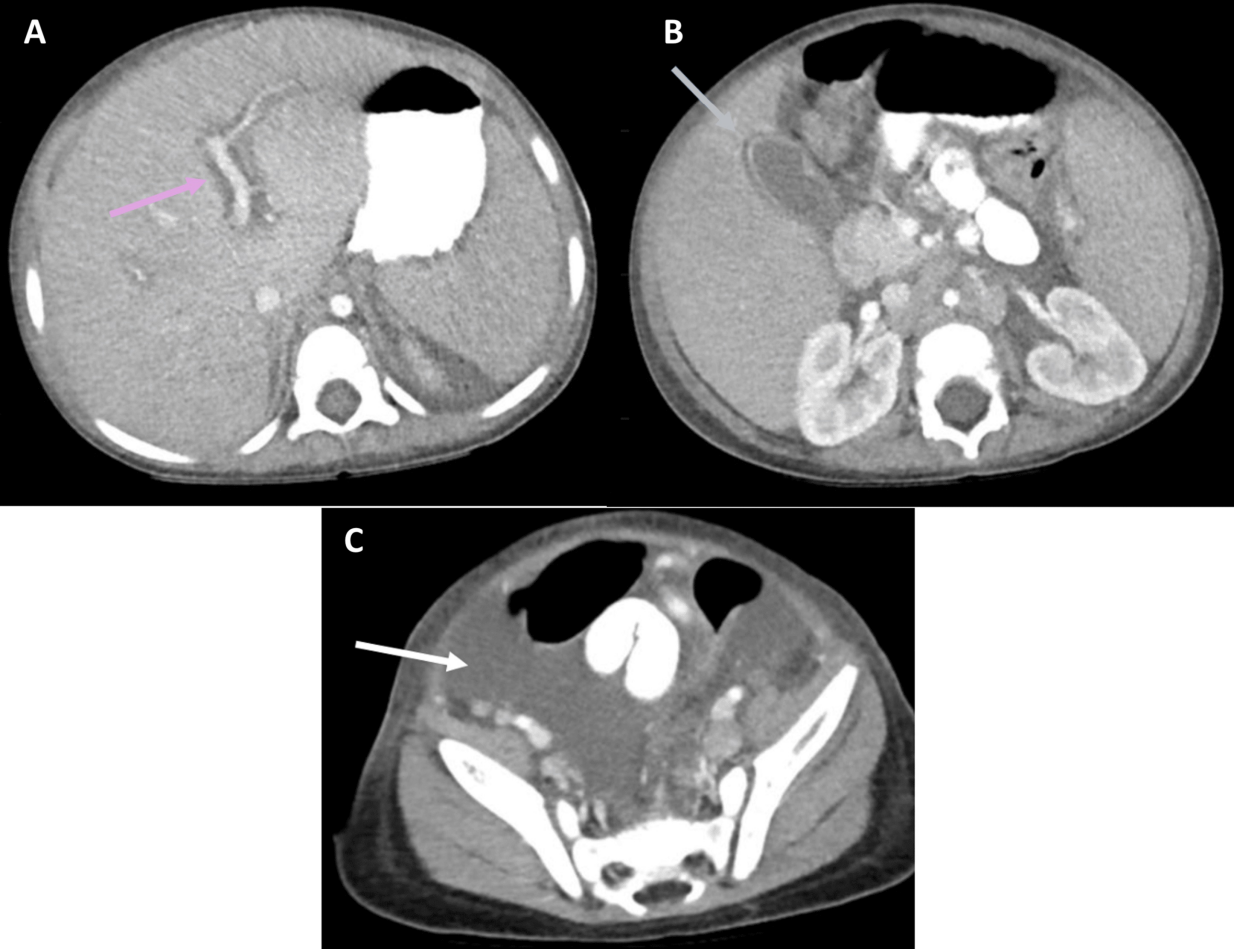
A two-year-old with HLH and multiorgan failure CT with IV contrast demonstrates peri-portal edema (arrow) (A), gallbladder wall thickening (arrow) (B), and ascites (arrow) (C). HLH: hemophagocytic lymphohistiocytosis

CT and MRI offer more precise visualization of abdominal abnormalities. The CT in Figure 3 is an example of what may help distinguish reactive lymphadenopathy due to inflammation from pathological enlargement due to an underlying malignancy. MRI provides high-resolution images without ionizing radiation [39]. It can be used to measure the liver and spleen size, as well as assess tissue composition [40]. Inflammation from HLH can lead to fibrosis, which may not be apparent on ultrasound or CT [41]. MRI is also effective for characterizing enlarged lymph nodes. In patients with chronic HLH, MRI can assess iron deposition in abdominal organs, which is a complication of prolonged inflammation and repeated blood transfusions. 

Neuroimaging

**Figure 4:**
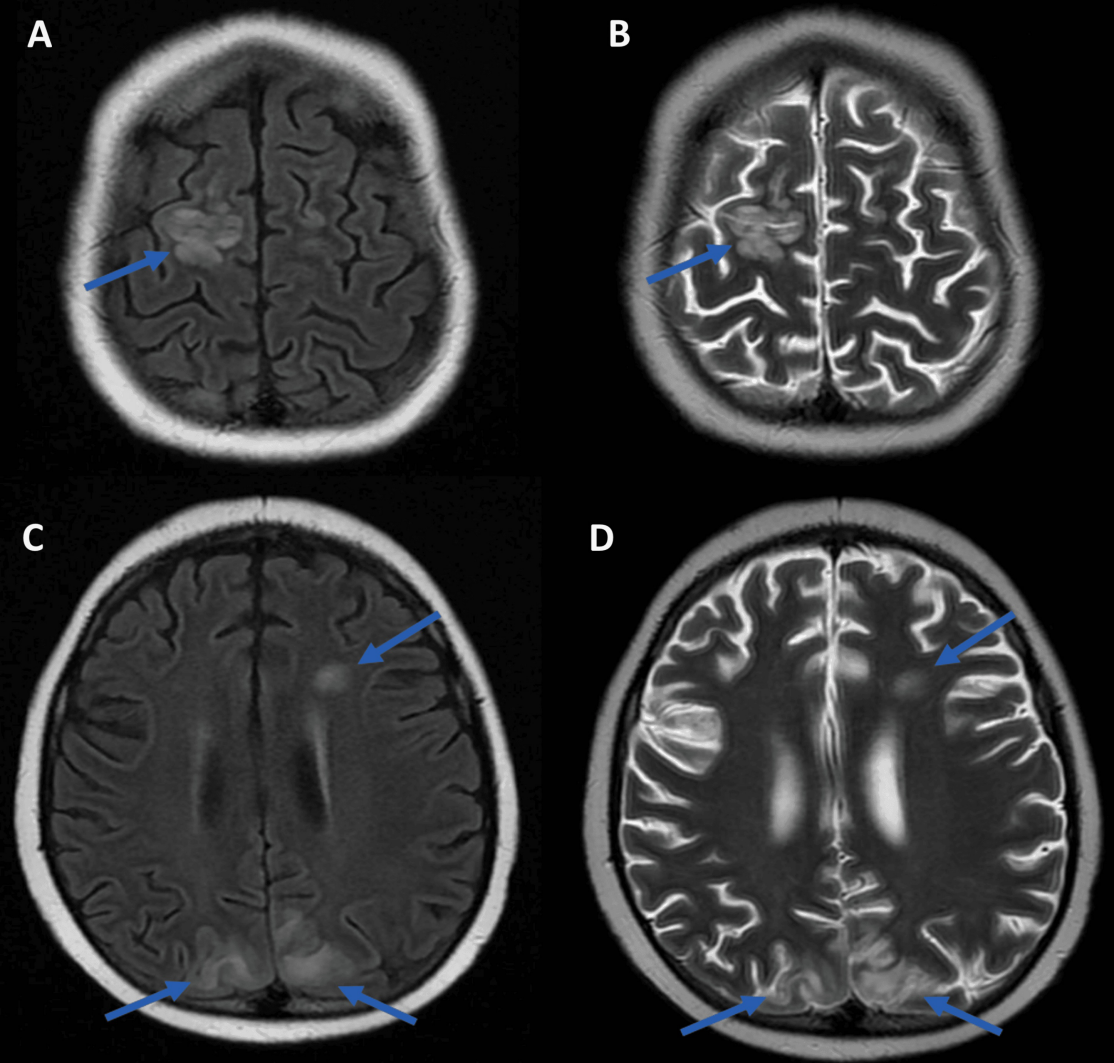
MRI images in a child with HLH and seizures FLAIR (A) and T2-weighted (B) brain MRI images in a child with HLH and seizures demonstrate multifocal hyperintense lesions (arrows in A and B) in the left frontal white matter and the right frontal (C) and biparietal cortical and subcortical regions (D) (arrows in C and D). HLH: hemophagocytic lymphohistiocytosis; FLAIR: fluid-attenuated inversion recovery

**Figure 5:**
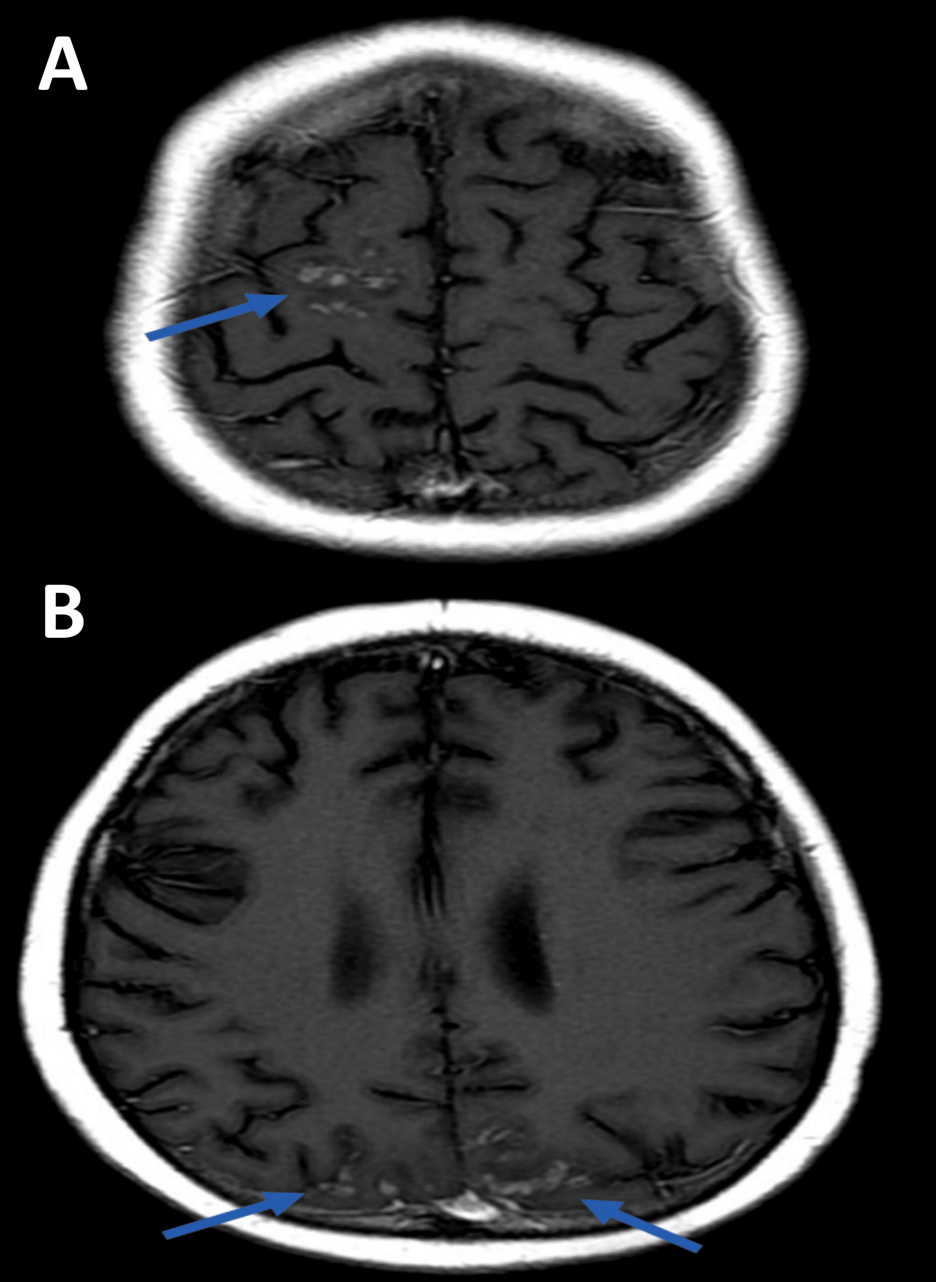
T1 post-contrast brain MRI images T1 post-contrast brain MRI images from the same child as Figure 4 demonstrate patchy enhancement in the right frontal and biparietal lesions (arrow) (A), but none in the left frontal lobe lesion (arrows) (B).

HLH involvement of the CNS can significantly impact clinical outcomes [42]. MRI is paramount in detecting CNS involvement, identifying subtle and overt abnormalities that may not be clear solely through history and physical examination (Figures 4, 5) [43]. One of the most important CNS findings for patients with HLH is hyperintensity on T2-weighted imaging [28,44,45]. This represents inflammation, edema, or demyelination in the brain and requires close monitoring. MRI may show T2/fluid-attenuated inversion recovery (FLAIR) hyperintense lesions in the white matter, cortical, and subcortical regions with variable enhancement [46-48]. 

While it is rare to capture hemophagocytosis on imaging, secondary signs of CNS involvement are common and useful in diagnosing and assessing CNS involvement in HLH [49]. These secondary signs include microhemorrhages and hemosiderin deposits, reflecting severe inflammatory damage within the CNS. Leptomeningeal enhancement can also be detected and typically manifests as headache, photophobia, and meningismus. For patients with chronic or severe HLH, MRI may show cerebral atrophy, which represents neuronal loss and long-term damage [44,45,50]. 

Clinical relevance

Radiologic findings in HLH are critical for several reasons. Ultrasound can be used for assessment of acute symptoms without exposing the patient to ionizing radiation [51]. CT and MRI offer more advanced diagnostic support and allow monitoring of CNS involvement as well as response to treatment [52]. Change in the hyperintensity of brain lesions or resolution of leptomeningeal enhancement can guide therapeutic decisions and enable clinicians to adjust treatment based on evolving imaging findings. Prognostic insights may be gained from imaging findings, which can help in counseling patients about the expected course of the disease and potential outcomes [27,50].

Treatment for hemophagocytic lymphohistiocytosis

Due to the varying etiology and presentation of HLH, treatment strategies may vary for the primary and secondary forms of the disease [24]. The main goal is to suppress any hyperactive immune responses and eliminate the underlying trigger [14]. In the case of primary HLH, replacing defective immune cells with healthy donor cells through hematopoietic stem cell transplantation is considered definitive [53]. The initial phase of treatment to control the immune response involves using a combination of immunosuppressive drugs and chemotherapy agents. Commonly used medications include corticosteroids, etoposide, and cyclosporine. Treatment guidelines have been standardized in the HLH-94 and HLH-2004 protocols [54]. These protocols detail an initial eight-week induction phase to achieve remission, followed by a continuation phase to prevent relapse [24]. 

Targeted therapy has also been used and shown to be effective in patients who are refractory to standard treatment or those who relapse after initial response to immunosuppression. These treatments inhibit the hyperinflammatory pathways involved in HLH [55]. Emapalumab inhibits IFN-γ activity and has been approved for use in patients with refractory, recurrent, or progressive HLH [56]. Ruxolitinib inhibits Janus kinase (JAK), interfering with the signaling pathway of multiple cytokines involved in HLH [57]. Its use as an off-label drug has shown promising results in reducing the disease progression [58]. 

Supportive treatment is also essential in the treatment of HLH. Due to the various cytopenias that are caused by HLH, blood transfusions are critical to prevent bleeding complications [59]. Intravenous immunoglobulin (IVIg) and antibiotics can be administered to treat infections, which may be the underlying trigger for HLH [60]. 

## Conclusions

Early diagnosis and treatment are essential in HLH, as both primary and secondary forms can progress rapidly and result in multiorgan failure. ⁤⁤Imaging, while not a diagnostic criterion for HLH, plays a crucial role in identifying organ involvement, monitoring disease progression, and guiding treatment decisions. As treatment options continue to evolve, a multidisciplinary approach, including imaging, will likely become increasingly important in HLH diagnosis and management. Furthermore, ongoing research into improved imaging techniques may enhance our ability to detect early organ involvement, contributing to more timely interventions and improved outcomes for HLH patients.
